# YO2 Induces Melanoma Cell Apoptosis through p53-Mediated LRP1 Downregulation

**DOI:** 10.3390/cancers15010288

**Published:** 2022-12-31

**Authors:** Yousef Salama, Satoshi Takahashi, Yuko Tsuda, Yoshio Okada, Koichi Hattori, Beate Heissig

**Affiliations:** 1 An-Najah Center for Cancer and Stem Cell Research, Faculty of Medicine and Health Sciences, An-Najah National University, P.O. Box 7, Nablus 99900800, Palestine; 2 Division of Clinical Genome Research, The Institute of Medical Science, The University of Tokyo, 4-6-1 Shirokanedai, Minato-ku, Tokyo 108-8639, Japan; 3 The Faculty of Pharmaceutical Science, Kobe Gakuin University, 518 Arise, Ikawadani-Cho, Nishi-Ku, Kobe 651-2180, Japan; 4 Center for Genome and Regenerative Medicine, Graduate School of Medicine, Juntendo University, 2-1-1 Hongo, Bunkyo-Ku, Tokyo 113-8421, Japan; 5 Department of Research Support Utilizing Bioresource Bank, Graduate School of Medicine, Juntendo University School of Medicine, 2-1-1 Hongo, Bunkyo-Ku, Tokyo 113-8421, Japan

**Keywords:** p53, miRNA, miR-103, mir-107, melanoma, LRP1, CD91, PLAT, PANK1

## Abstract

**Simple Summary:**

We found that induction of tumor suppressor p53 in melanoma cells suppressed tumor growth by targeting LRP1 expression. LRP1 downregulation was achieved by the micro RNAs miR103/107. We identify the small molecule YO-2 as an inducer of tumor suppressor p53. We propose that YO-2, combined with myelosuppressive drugs like doxorubicin, can be a novel treatment option.

**Abstract:**

The multifunctional endocytic receptor low-density lipoprotein receptor-related protein 1 (LRP1) has been implicated in melanoma growth. However, the mechanism of LRP1 expression in melanoma cells remains only partially understood. In most melanomas, the TP53 tumor suppressor is retained as a non-mutated, inactive form that fails to suppress tumors. We identify TP53 as a regulator of LRP1-mediated tumor growth. TP53 enhances the expression of miRNA miR-103/107. These miRNAs target LRP1 expression on melanoma cells. TP53 overexpression in human and murine melanoma cells was achieved using lentivirus or treatment with the small molecule YO-2, a plasmin inhibitor known to induce apoptosis in various cancer cell lines. TP53 restoration enhanced the expression of the tumor suppressor miR-103/107, resulting in the downregulation of LRP1 and suppression of tumor growth in vivo and in vitro. Furthermore, LRP1 overexpression or p53 downregulation prevented YO-2-mediated melanoma growth inhibition. We identified YO-2 as a novel p53 inducer in melanoma cells. Cotreatment of YO-2 with doxorubicin blocked tumor growth in vivo and in a murine melanoma model, suggesting that YO-2 exerts anti-melanoma effects alone or in combination with conventional myelosuppressive drugs.

## 1. Introduction

Although melanoma accounts for only ~1% of all skin cancers in the U.S., it is the deadliest form of the disease. Treatments such as immunotherapy combining anti-CTLA4 and anti-PD-1 checkpoint inhibition have pushed the 5-year relative survival for advanced-stage disease from 15% in 2004 to 30% for patients diagnosed from 2011 through 2017 [1]. However, these data indicate the urgent demand to search for more effective or alternative treatment strategies and the necessity to elucidate the associated molecular mechanisms.

The low-density lipoprotein receptor-related protein 1 (LRP1/CD91) is a transmembrane endocytic receptor. LRP1 augments melanoma growth and metastasis by binding to the fibrinolytic factor tissue-type plasminogen activator (tPA) [2]. Restoration of LRP1 in the less aggressive, poorly metastatic B16F1 tumor cells enhanced tumor cell proliferation and led to lung metastasis in murine melanoma models. Yiong et al. reported that yes-associated protein promotes tumorigenesis in melanoma cells through LRP1 stimulation [3]. The cancer-secreted LRP1 ligand, Apolipoprotein E (ApoE), suppresses invasion and metastatic melanoma cells through LRP1 [4]. However, the exact mechanism of how LRP1 expression is regulated in melanoma cells remains undetermined.

TP53, often referred to as p53, restricts proliferation in response to DNA damage by inducing cell cycle checkpoints, apoptosis, or cellular senescence. Although 80% of melanoma tumors retain non-mutated, wild-type TP53 expression [5], TP53 fails to function as a tumor suppressor in melanoma cells, and reduced levels of p53 contribute to aggressiveness and resistance to therapy. A recent study demonstrated that TP53 targets the translation of LRP1 and promotes cell death by inducing the microRNAs (miRNAs) miR-103 and miR-107 after the induction of lethal stress [6].

MicroRNAs are a class of small molecules that regulate gene expression. The pre-miR-107 sequence is located within an intron of the *pantothenate kinase1 (PANK1)* gene. MiR-103 and miR-107 are related miRNAs that differ by only one base in their 3` regions [7]. p53 activates the *PANK1*/*miRNA-107* gene leading to the downregulation of CDK6 and p130 cell cycle proteins [8]. miR-107 is downregulated in metastatic melanoma. A recent study demonstrated that miR-107 is a novel tumor-suppressive factor in melanoma [9]. miR-107 overexpression (OE) reduced melanoma cell proliferation, migration, and invasion.

Recent studies indicate that plasminogen activators such as tPA (also known as PLAT) and plasminogen (plg) receptors are TP53 targets that accelerate tumor growth/metastasis [10]. The small-molecule plasmin inhibitor YO-2 induces apoptosis in different hematopoietic and solid cancer cell types [11,12,13]. In addition, we observed that YO-2 treatment downregulated LRP1 in melanoma cells [2].

Herein, we studied the molecular mechanism of LRP1 downregulation by YO-2 that induces apoptosis in melanoma cells. We identified a novel regulatory circuit necessary for melanoma cells that involves LRP1, TP53, and miR103/107. We show that YO-2 induces melanocyte apoptosis through the upregulation of TP53. We found that the apoptosis-inducing effects of YO-2 were mediated through LRP1 as restoration of LRP1 rendered melanoma cells YO-2 resistant. Mechanistically, YO-2 upregulated TP53 that restored miR103/107 expression, which in turn targeted expression of LRP1, a receptor necessary for melanoma cell proliferation. YO-2 might improve the anti-tumor efficacy of myelosuppressive regiments.

## 2. Materials and Methods

### 2.1. Mice

We used 8- to 12-week-old male WT C57BL/6 mice (An-Najah National University, Nablus, Palestine) for the experiments. The Institutional Animal Care and Use Committee of An-Najah National University approved the animal procedure protocols.

### 2.2. Cell Lines

The B16F10 (CRL-6475; American Type Culture Collection (ATCC), Manassas, VA, USA) and B16F1 (CRL-6323) melanoma cell lines were cultured in high-glucose Dulbecco’s Modified Eagle’s Medium (DMEM with L-glutamine, phenol red (Fuji Film Wako, Osaka, Japan), 10% fetal bovine serum (FBS; G.E. Healthcare, Chicago, IL, USA), and 1% penicillin/streptomycin (P/S) (Nacalai Tesque, Kyoto, Japan)). In addition, RPMI-8226 and B53 cells were cultured in RPMI 1640 (4500 mg/L glucose) containing 2 mM glutamine and 10% FBS. Mouse embryonic fibroblast-1 (MEF-1) cells were cultured in high-glucose DMEM containing 10% FBS and 1% P/S. The human melanoma cell line SK-MEL-28 (ATCC HTB-72) and the human epidermoid carcinoma cell line A431 (ATCC CRL-1555) were expanded in Eagle’s Minimum Essential Medium containing 10% FBS. Human umbilical vein endothelial cells (HUVECs) were cultured on 0.1% gelatin (Wako Pure Chemicals)-coated culture plates (Falcon) in endothelial growth medium-2 (EGM-2; Lonza; Tokyo, Japan, cc4176). The T17B cell line was maintained in DMEM, 10% FBS, and 1% P/S.

The human Hs 688 (A).T (ATCC; CRL-7425) cells—a human melanoma cell line—were cultured in DMEM, 10% FBS, and 1% P/S. Peripheral blood mononuclear cells were isolated from heparinized blood of a healthy donor. Mouse embryonic fibroblasts were cultured in high glucose DMEM, 10% FBS, and 1% PS.

### 2.3. Cell Culture

We transfected B16F10 cells (1 × 10^5^ cells/well) by using Lipofectamine RNAiMAX (Invitrogen, Waltham, MA, USA). B16F10 wild-type, LRP1 or p53 overexpressing (OE) or knockdown (KD) cells, SK-MEL-28, or A431 cells (1 × 10^5^ cells/well) were seeded in triplicate in 6-well plates (Thermo Fisher Scientific, Lafayette, CO, USA) and were left for 16 h overnight to attach before the treatment started. We treated cells with/without YO-2, or DMSO/PBS controls for 24 h. In some experiments, B16F10 cells were cultured with/without rec. tPA (1500 U/mL) and rec. plasminogen (100 ng/mL). Trypan blue negative cells were determined as viable cells (cat. 207-17081; Fuji Film Wako). We used the LDH assay kit (ab65393, Abcam, Cambridge, UK) to determine cytotoxicity and the Caspase 3/7 activity assay (Promega, Tokyo, Japan) to assess caspase 3 and 7 activity as recommended by the manufacturers.

Cell lines were cultured for 16 h. Then, cells were exposed to UV at 254 nm using UV lamp VL-6.LC (Vilber Lourmat, Eberhardzell, Germany).

### 2.4. Small Interfering Ribonucleic Acids (siRNA)-Based Gene Knockdown

The 2 × 10^5^ cells/well were plated in a 6-well plate for 16 h before transfection using siRNA targeting LRP1, human p53, miR-107, or control sequences (Invitrogen; Thermo Fisher Scientific, Lafayette, CO, USA). siRNAs were transfected into cancer cells at a final concentration of 100 nM using Lipofectamine™ RNAiMAX Transfection Reagent (Invitrogen). The transfection media was removed after 12 h. Then, cells were cultured in fresh media. The following siRNAs were used:
**SiRNAs****Sequences of the Synthesized Oligonucle Otides**si-miR-1075′-GCAUUGUACAGGGCUAUCAAA-3′si-LRP1#15′-GCUCAUCUCGGGCAUGAUU-3′si-LRP1#25′-GCAGUUUGCCUGCAGAGAUUU-3′Si-Ctrl5′-GCUCCACAGAGUAUACCUU-3′Si-hu p53 was purchased from Santa Cruz as a Lipofectamine RNAi/Max kit (cat no.-29435). We quantified the transfection efficiency using qPCR and Western blotting of the target gene in Si-target gene and Si-ctrl transfected cells.


### 2.5. In Vivo B16F10 Melanoma Model

Local tumor model: B16F10 cells (90% viability determined by trypan blue exclusion) were inoculated on d 0 (1 × 10^6^/200 μL/mouse, s.c.) into C57/BL6 mice. Mice were euthanized when they showed severe pain, had bodyweight loss > 20% compared to the initial body weight, or appeared moribund. We evaluated the tumor growth daily. The animals were euthanized by cervical dislocation, and tumors and adjacent conjunctive tissues were removed twelve days after subcutaneous and intravenous tumor cell inoculation. Extracted tumors were weighed at d 12.

### 2.6. In Vivo Drug Treatment Scheme

YO-2 (5 mg/kg bodyweight—if not otherwise indicated; kindly provided by Yuko Tsuda, Osaka University, Osaka, Japan) or carrier (DMSO/PBS) was injected intraperitoneally daily starting on day 5 after tumor cell inoculation. In addition, in some experiments, mice were treated with doxorubicin (1 mg/kg bodyweight as a single dose on day 0 (Sigma, Tokyo, Japan) alone or cotreated with YO-2 starting from day 5 post-injection.

### 2.7. Quantitative Reverse Transcriptase-Polymerase Chain Reaction (qPCR)

RNA isolation was conducted as previously described [2]. In brief, total RNA was extracted from cell lines using Trizol reagent and reverse-transcribed into complementary DNA (cDNA) using the PrimeScript RT Master Mix. The miRNeasy Mini Kit (Qiagen, Hilden, Germany) was used to extract miRNA. According to the manufacturer’s instructions, reverse-transcription was performed using the Mir-XTM miRNA First-Strand Synthesis Kit (cat no 638313, Takara, Tokyo, Japan).

Gene expression was determined by comparative SYBR green qPCR using an Applied Biosystems StepOnePlus Real-time qPCR. We calculated the relative mRNA expression for all genes and miRNAs using the 2^−ΔΔCt^ method with b-actin mRNA as an internal normalizer unless otherwise mentioned. The levels of expression of mi-103 and miR-107 were normalized to U-6 mRNA. Each qPCR experiment was performed in triplicate and independently repeated two times. The respective forward and reverse primers used were:
**Gene****Forward Primer****Reverse Primer**mPUMA5′-CCTGGAGGGTCATGTACAATCT-′35′-GTTGGGCTCCATTTCTGGGG-′3mBAX5′-AGGCCTCCTCTCCTACTTCG-′35′-GTGAGGACTCCAGCCACAAA-′3mBCL25′-CCACCTGTGGTCCATCTGAC-′35′-ATCTCTGCGAAGTCACGACG-′3mLRP15′-GGACCACCATCGTGGAAA-‘35′-TCCCAGCCACGGTGATAG-‘3mp535′-GCCCATGCTACAGAGGAGTC-′35′- GGGAAGTAGACTGGCCCTTC-′3mp215′-TTGTCGCTGTCTTGCACTCT-′35′-TTTCGGCCCTGAGATGTTCC-′3mPANK15-TCAGCAAAGAAGACCTCGCC-′35-GTGCACACATCCGAGCAATG-′3mβ-actin5′-CTAAGGCCAACCGTGAAAAG-′35′-ACCAGAGGCATACAGGGACA-′3hup535-GGCCCATCCTCACCATCATC-′35-CACGCACCTCAAAGCTGTTC-′3huLRP15′-GATGAGACACACGCCAACTG-‘35′-CGGCACTGGAACTCATCA-‘3huPANK15′-TTTCCCAGCTGTGCTATGCA-′35′-AGGGTGGTGTGAAGGCTAGA-′3huβ-actin5′-CCAACCGCGAGAAGATGA-‘35′-CCAGAGGCGTACAGGGATAG-‘3h-U6-FCGCTTCGGCAGCACATATAC
h-U6-R and mU6F mRQ 3′ Primer from Mir-X miRNA First-Strand Synthesis Kit (Takara, cat no. 638313)m-U6-FCTCGCTTCGGCAGCAAC
mu-miR-1035′-AGCAGCATTGTACAGGGCTATGA-′3kit MRQ smart (Takara, Japan)Hu-miR-1035′-AGCAGCATTGTACAGGGCTATGA-′3kit MRQ smartHu-miR-1075′-AGCAGCATTGTACAGGGCTATCA-′3kit MRQ smartMu-miR-1075′-AGCAGCATTGTACAGGGCTATCA-′3kit MRQ smart

### 2.8. LRP1 Lentivirus Generation for LRP1 OE

LRP1 cloning as previously described [2].

### 2.9. Hu P53 Cloning

We used A549 human lung cells to clone the human P53 coding sequence into the LV-EF-L3T4-IRES2-EGFP vector. The P53 cloning sequence was: Restriction EnzymeP53 Coding SequenceXhoI5-GGGCTCGAGATGGAGGAGCCGCAGTCAGA-3SmaI5-CCCCCCGGGTCAGTCTGAGTCAGGCCCTTC–3

### 2.10. miR-107/103 Cloning

Genomic DNA from HUVEC cells was extracted using the genomic DNeasy Extraction Kit (Qiagen, Germany). PrimeSTAR polymerase (Takara, Japan) was used to amplify the genomic sequence by PCR. The following primers were used:
miR-1075`-GGGCTCGAGAAGCAGGCTAAAATTCCAGTC-3`miR-1075-CCCCCCGGGCCCAAAAGAACTTAGCAATCTT-3`miR-1035`-GGGCTCGAGAACTGTTGAAAAGGACACTGTGG-3`miR-1035`-CCCCCCGGGCAGCAGCCACTGAGCCATTTCCC-3`

The purified amplicons were inserted into the XhoI and SmaI sites of the eukaryotic expression vector LV-EF-L3T4-IRES2-EGFP. Following Sanger sequencing (An-Najah University, Nablus, Palestine), cloned plasmids were amplified in *E. coli* DH5α competent cells.

### 2.11. Purchased Plasmids

The murine P53 KD pLenLox_U6P53 (Plasmid #59360, Addgene, Watertown, MA, USA) [14] and the murine P53 OE pMXs-P53 (Plasmid #22725; Addgene) were included [15].

### 2.12. Lentivirus Production

Vesicular stomatitis virus glycoprotein–pseudotyped lentivirus was prepared using a three-plasmid system: the plasmid LV-EF-L3T4-IRES2-EGFP (kindly provided by Dr. Trono’s laboratory and modified by Dr. Tomoyuki Yamaguchi, IKAKEN) with or without gene of interest, pMDL, and vesicular stomatitis virus glycoprotein envelope plasmid VSV.G (Addgene #14886). The three plasmids were co-transfected into 293T cells using polyethylenimine (Takara, Japan). Viral supernatant collection was done 48 h later. Virus aliquots were stored at −80 °C. The viral titer was determined using 293T cells.

### 2.13. Western Blot Analysis

Protein crude lysates recovered after the addition of Cell Lysis Buffer (10×; #9803, Cell signaling) and protease inhibitors (Complete Mini, Roche, Basel, Switzerland) were centrifuged at 14,000× *g* for 10 min at 4 °C. We used BSA protein to determine the protein concentration of the supernatants (Bio-Rad Laboratories, Hercules, CA, USA). We applied cell lysates (2–50 μg protein) on 12% acrylamide gel. Following protein transfer to a PVDF membrane (Immobilon, Millipore, Burlington, MA, USA), the following primary antibodies (all mouse IgG, 1 µg/mL) overnight at 4 °C: p53 of mouse origin (sc-126, Santa Cruz Biotech, Dallas, TX, USA), LRP-1 (sc-25469, Santa Cruz Biotech), b-actin (4967S, Cell Signaling Technology, Danvers, MA, USA) were used to stain the membranes. We stained the membranes with secondary antibody conjugated with horseradish peroxidase (rabbit-HRP, or mouse-HRP, Nichirei Biosciences Inc., Tokyo, Japan) and developed them with the ECL Plus detection system (RPN2132, Amersham Life Science). Images were taken using image analyzer C-280 Azure (Azure Biosystems Inc., Dublin, CA, USA).

### 2.14. Webserver Timer

As reported previously, here we also used the web server TIMER to analyze the gene expression across diverse cancer types (http://gepia2.cancer-pku.cn/#index), accessed on 26 November 2022 [16].

### 2.15. Statistical Analysis

Experiments were performed three times if not otherwise noted. Data are presented as the mean +/− standard error of the mean (SEM). The statistical analysis included the following tests: Student’s *t*-test or ANOVA with Tukey HSD post hoc tests using R program. *p*-values < 0.05 were considered statistically significant.

## 3. Results

### 3.1. YO-2 Induces Cell Apoptosis in Melanoma Cells

**Figure 1:**
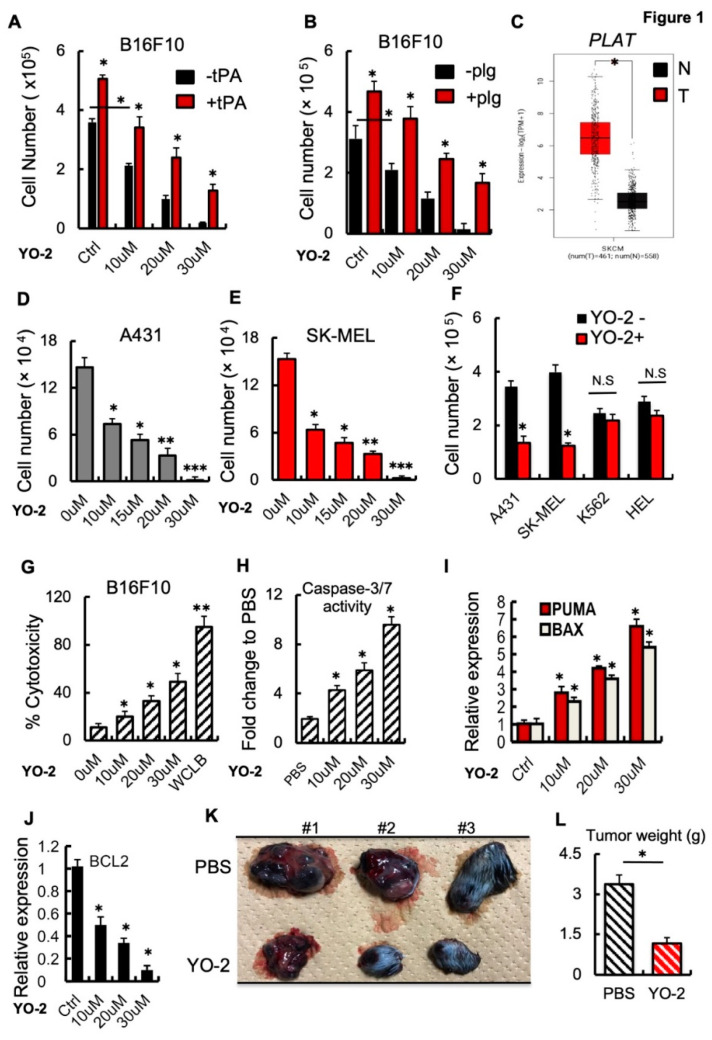
YO-2 induces apoptosis in melanoma. (**A**,**B**) Cultured B16F10 cells were treated with/without rtPA (**A**) or plasminogen (plg) (**B**) and cotreated with the indicated concentration of the plasmin inhibitor YO-2. Cells were counted after 24 h (*n* = 6). (**C**) Human *tPA* expression levels in tumor (T) and normal adjacent (N) tissues of human skin cutaneous melanoma (SKCM) patients were retrieved from the TCGA database and analyzed by Timer. (**D**,**E**) Human A431 (**D**) and SK-MEL-28 (**E**) skin cancer cells were cultured with/without various concentrations of YO-2. Viable cells were enumerated after 24 h *n* = 6). (**F**) Indicated cell lines (human A431 and SK-MEL-28 melanoma or myeloid K562 and HEL cells) were cultured in the presence or absence of YO-2 (10 μM/mL). Viable cells were counted after 24 h (*n* = 6). (**G**) The LDH assay kit was used to determine cytotoxicity in B16F10 cells treated with/without YO-2 after 24 h (*n* = 6). (**H**) Caspase 3/7 activity in supernatants of B16F10 cells treated with/without YO-2 (*n* = 6). (**I**,**J**) Fold change in apoptosis-linked *PUMA* and *BAX* (**I**) and the anti-apoptosis-linked *BCL2* (**J**) gene expression in YO-2-treated and control B16F10 cells by qPCR (*n* = 3). Expressions were compared to untreated/carrier cell controls. (**K**,**L**) B16F10 wild-type tumor-bearing mice were treated with/without the plasmin inhibitor YO-2 starting from day 5. On day 12, representative macroscopic tumor images were taken (**K**), and the tumor weight was determined (**L**; *n* = 6). Data are expressed as mean ±  SEM (unpaired Student’s *t*-test or one-way ANOVA * *p* < 0.05, ** *p* < 0.01, *** *p* < 0.001).

We observed that the small-molecule plasmin inhibitor YO-2 downregulates LRP1 expression and suppresses melanoma growth [2]. To test whether YO-2 can block tPA-mediated melanoma cell growth, the murine melanoma cell line B16F10 was treated with recombinant (rec.) tPA in the presence of YO-2. The addition of the plasmin inhibitor YO-2 prevented tPA and plasminogen-induced proliferation of the murine melanoma cell line B16F10 in a dose-dependent fashion (Figure 1A,B). *tPA* expression in patients with skin cutaneous melanoma (SKCM) was higher in tumor (T) compared with adjacent normal (N) skin tissues (Figure 1C). YO-2 inhibited cell growth in human A431, SK-MEL, and murine B16F10 melanoma cells (Figure 1D–F). YO-2 did not alter cell proliferation of the myeloid K562 and HEL cells at a concentration of 10 μM, while this dose had been effective in controlling melanoma cell growth (Figure 1F). The release of lactate dehydrogenase was measured in YO-2-treated B16F10 culture supernatants to assess plasma membrane damage and cytotoxicity level. While the addition of the positive control (whole cell lysis buffer, WCLB) caused cytotoxicity in 100% of treated cells, YO-2 at a concentration of 30 μM induced cytotoxicity in ~50% of treated B16F10 cells (Figure 1G). Caspases result in the cleavage of protein substrates, causing the disassembly of the cell. YO-2 treatment increased the cleaved forms of caspase-3 and caspase-7, suggesting that the canonical apoptosis program was activated (Figure 1H).

Next, we examined the effect of YO-2 on the expression of the pro-apoptotic genes Puma and Bax in cultured B16F10 cells. The transcript expression levels of *Puma* and *Bax* increased with increasing concentrations of YO-2 (Figure 1I). In contrast, the apoptosis-inhibiting BCL2 gene was downregulated in YO-2-treated B16F10 cells (Figure 1J). Establishment of a B16F10 subcutaneous melanoma model provided additional support for the effectiveness of YO-2 in controlling melanoma growth. YO-2 treatment started five days after the initial tumor inoculation. Compared to the control (DMSO), YO-2 treatment dramatically inhibited tumor growth (Figure 1K,L).

### 3.2. YO2 Downregulates LRP1 Expression on Melanoma Cells

**Figure 2:**
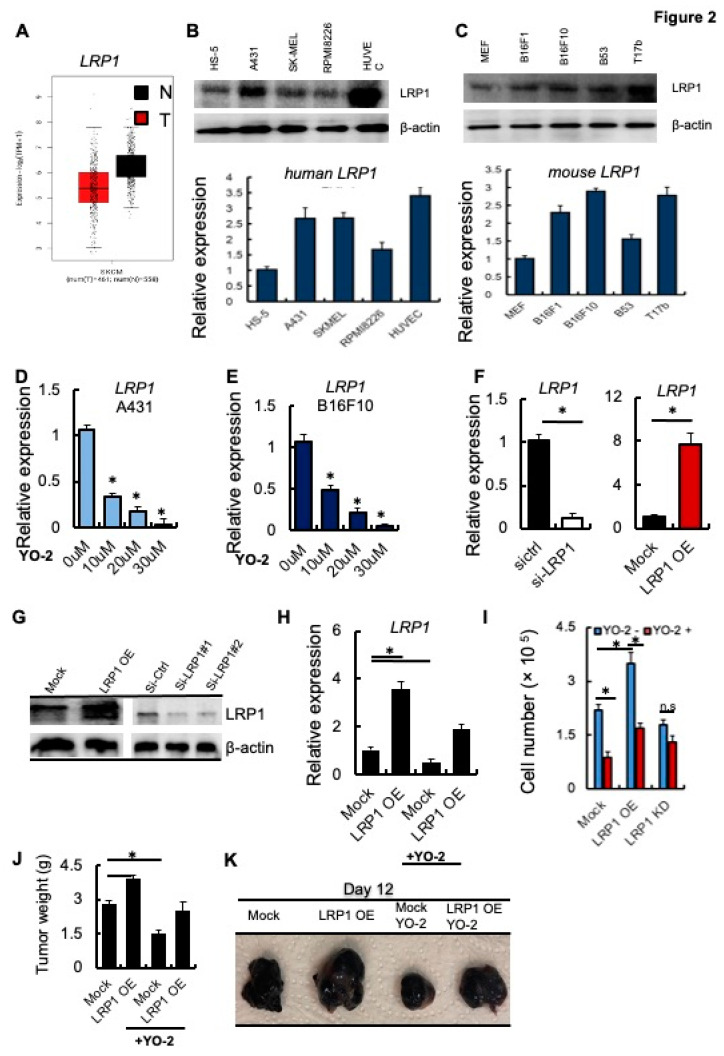
YO-2 modulates melanoma cell growth through LRP1 downregulation**.** (**A**) Human *LRP1* expression levels in tumors (T) and normal tissues (N) of SKCM patients were retrieved from the TCGA database and analyzed by Timer. (**B**,**C**) Top panels: Representative immunoblots for LRP1 and b-actin. Bottom panels: Human HS-5, A-431, SK-MEL-28, RPMI8226, HUVEC and murine MEF, B16F1, B16F10, B53, and T17b cells (**C**) were analyzed for *LRP1* mRNA expression by qPCR (*n* = 3/group). Fold change of expression was determined, and expression was compared to untreated cell controls. (**D**,**E**) Fold change in *LRP1* expression was determined in human A431 (**D**) and mouse B16F10 (**E**) cells treated with different concentrations of YO-2 (*n* = 3). The expression was compared to untreated cell controls. (**F**) Fold change in *LRP1* transcript levels determined by qPCR (**F**) in si-RNA gene silenced si-LRP1 or si-ctrl and Mock or LRP1 OE cells. The expression was compared to si-ctrl/Mock controls. (**G**) Representative immunoblots for LRP1 and b-actin (loading controls) using mock, LRP1 OE, and si-ctrl or si-LRP1#1 and si-LRP1#2 cell lysates. (**H**) Fold change in *LRP1* expression of mock or LRP1 OE cells treated with/without YO-2 (*n* = 3). The expression was compared to untreated mock controls without YO-2 treatment. (**I**) Proliferation of Mock, LRP1 KD, or LRP-1 OE B16F10 cells treated with/without YO-2 after 24 h in culture. (**J**,**K**) Mock, LRP1 OE B16F10 cells were injected subcutaneously into C57BL6 mice. Groups of mice were treated with/without YO-2 starting from day 5. Twelve days after inoculation, the tumor weight was measured (**J**; *n* = 6/group), and representative macroscopic tumor images were taken (**K**). Data are expressed as mean ± SEM (unpaired Student’s *t*-test or one-way ANOVA * *p* < 0.05; n.s.).

Several studies indicate that LRP1 controls melanoma cell growth [2,3]. We analyzed the expression of LRP1 in tumor tissues of SKCM patients (T) and healthy controls (N) using TIMER. The *LRP1* expression was lower in the tumor than in adjacent normal human skin melanoma tissues (Figure 2A). The human skin carcinoma and melanoma cells A431 and SK-MEL-28 or the murine B16F1 and B16F10 (high and low metastatic potential) showed impaired *LRP1* expression compared to fibroblast/stromal cell controls (HS-5 and murine MEF1 cells). Human and murine plasma or endothelial cell lines showed high *LRP1* transcript levels as determined by Western blotting and qPCR (Figure 2B,C). 

Next, we examined the transcript levels of *LRP1* in response to 10, 20, and 30 μM YO-2 treatment. YO-2 dose-dependently decreased LRP1 expression in A431 and B16F10 cells (Figure 2D,E), suggesting that YO-2 impairs LRP1 expression in melanoma cells. We established B16F10 cells where we gene-silenced LRP1 (si-LRP1) or overexpressed LRP1 using a lentivirus (LRP1 OE). We confirmed LRP1 expression by qPCR and Western blotting (Figure 2F,G). YO-2 treatment of Mock and LRP1 OE cells reduced *LRP1* expression (Figure 2H). Consistent with the role of LRP1 in melanoma cell growth, we found that LRP1 OE enhanced melanoma cell growth (Figure 2I). YO-2 treatment reduced melanoma proliferation in Mock and LRP1 OE expressing, but not LRP1 KD cells (Figure 2H,I) in vitro, and tumor growth in Mock and LRP1-OE tumors in vivo (Figure 2J,K). These data indicate that the antiproliferative effects of YO-2 were linked to cellular LRP1 expression.

### 3.3. p53 Is Required for YO-2-Driven LRP1 Expression

**Figure 3:**
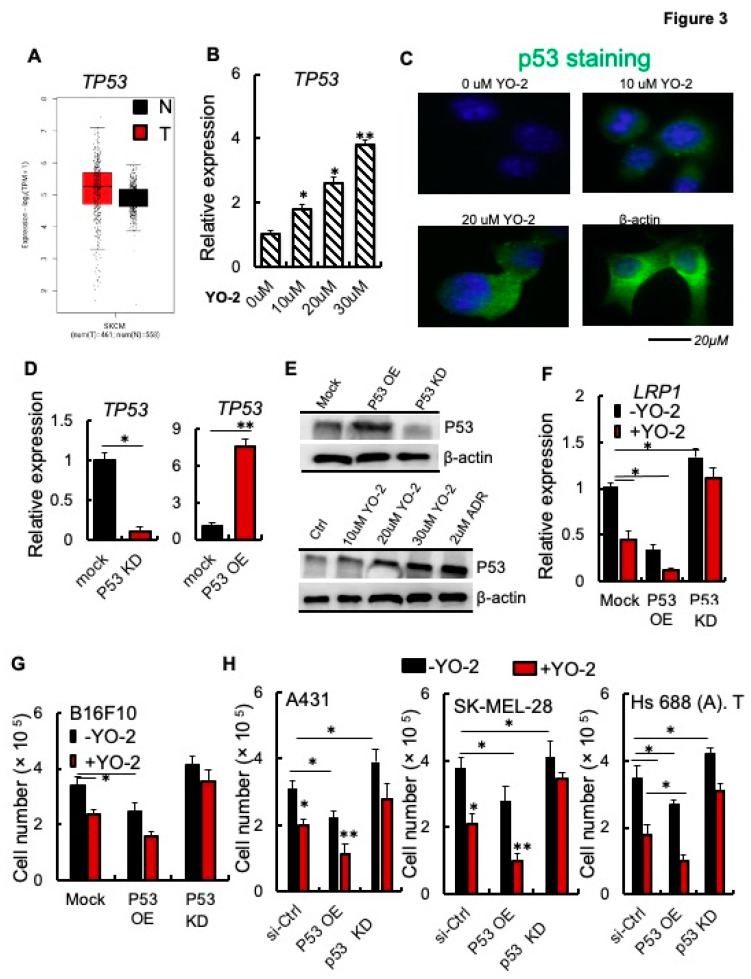
p53 is required for the antiproliferative effects of YO-2 in melanoma cells**.** (**A**) Human *TP53* expression levels in tumor (T) and adjacent normal tissues (N) derived from SKCM patients. Data were retrieved from the TCGA database and analyzed by Timer (n.s.). (**B**) *TP53* expression was determined in mouse B16F10 cells treated with different concentrations of YO-2 (*n* = 3). The expression was compared to untreated cell controls. (**C**) Representative immunofluorescence images of p53-stained B16F10 cells treated with or without indicated concentrations of YO-2. (**D**) Fold change *in TP53* expression as compared to mock/controls in B16F10 cells overexpressing murine p53 (P53 OE) or in cells with si-p53 knockdown (P53 KD). Expression was determined by qPCR (*n* = 3/group/experiment). Expression was compared to untreated controls. (**E**) Representative immunoblots for murine TP53 using lysates from mock, P53 OE, P53 KD cells (upper panel), or cell lysates from B16F10-tumor-bearing mice treated with or without YO-2 at the indicated concentrations, doxorubicin (2 μM). b-actin served as a loading control. (**F**) Fold change in *LRP1* expression in P53 OE and P53 knockdown (KD) compared to mock B16F10 cells after treatment with/without YO-2 (10 μM) and 24 h in culture (*n* = 3/group). (**G**) Cell proliferation of P53 OE and P53 KD B16F10 cells treated with/without YO-2 (*n* = 6/group). (**H**) Si-ctrl, P53 OE, and P53 siRNA gene silenced human A431, SK-MEL-28, and Hs 688 (**A**). T cells were treated with or without YO-2 (*n* = 6/group). Cells were counted 36 h after initial plating. Data are expressed as mean ± SEM (unpaired Student’s *t*-test or one-way ANOVA * *p* < 0.05, ** *p* < 0.01).

Recently, LRP1 has been identified as a downstream target of the tumor suppressor gene p53 (also called TP53) in MEF1 and colon cancer cells [6]. *TP53* is not differentially expressed in malignant melanoma cells (T) and normal (N) skin tissues of patients with SKCM (Figure 3A). We determined TP53 expression after YO-2 exposure in melanoma cells. The wild-type, unmuted form of TP53 is prevalent in melanoma cells. The murine cell line B16F10 maintains functional wild-type TP53. Thus, we examined if YO-2 augmented TP53 transcript and protein expression, which indeed was the case as shown by qPCR and immunohistochemistry (Figure 3B–D). Next, we established B16F10 cells in which we knocked down p53 (P53 KD) or overexpressed TP53 using a lentivirus (P53 OE). We confirmed TP53 expression by qPCR and Western blotting (Figure 3D,E, upper panel). TP53 protein was upregulated in B16F10 cells following treatment with various YO-2 doses compared to untreated control cells (Figure 3E, lower panel). The well-established TP53-inducing drug doxorubicin served as a positive control.

We studied LRP1 expression in cells with varying expression levels of TP53. TP53 KD augmented, while P53 OE impaired *LRP1* expression compared to mock B16F10 melanoma cells (Figure 3F). YO-2 treatment reduced LRP1 expression in mock and pP53 OE but not P53 KD cells indicating that YO-2-mediated LRP1 downregulation involved p53 (Figure 3F). P53 KD in B16F10 cells was achieved after introducing an shRNA targeting TP53 using a lentivirus, while P53 KD in the human cell lines was achieved using siRNA targeting TP53. YO-2-mediated cell proliferation suppression depended on the TP53 status of the tumor cells, as shown using murine B16F10 (Figure 3G), and the human skin cancer A431, SK-MEL-28, and Hs 688 (A). T cells (Figure 3H). P53 OE suppressed, while P53 KD enhanced tumor growth (Figure 3G,H). YO-2 impaired tumor growth in mock and P53 OE in all cell lines, but not P53 KD or P53 siRNA KD cells (Figure 3G,H). These data suggest that YO-2 mediates its antiproliferative effects through the upregulation of TP53.

### 3.4. YO-2-Induced miR-107/miR-103 Downregulate LRP1 in Melanoma Cells

**Figure 4:**
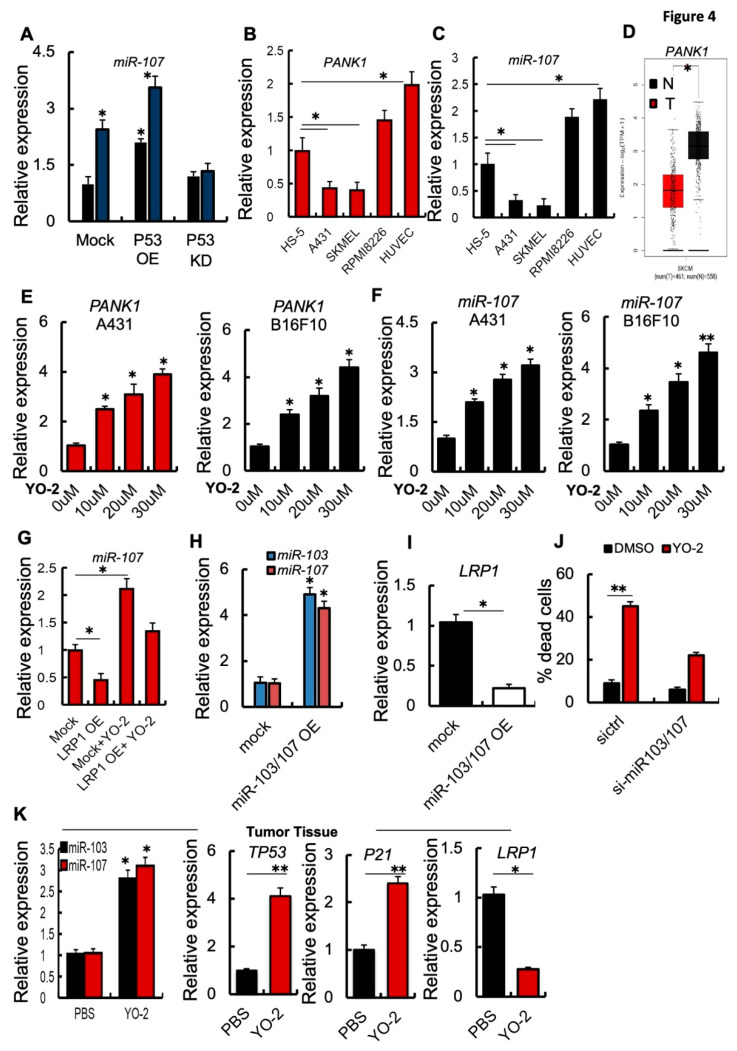
YO-2 restores tumor suppressor miR-107/103 expression. (**A**) Fold change in *miR-107* expression in P53 OE and P53 KD murine B16F10 cells treated with/without 10 μM/mL YO-2 following a 24 h culture period. In addition, expressions were compared to untreated Mock controls. (**B**,**C**) Fold change in human *PANK1* and *miR-107* expression in HS-5, A431, SK-MEL-28, and HUVEC cells. The expression was compared to the expression in HS-5 cells (*n* = 3/cell line). (**D**) Human *PANK1* expression levels in tumors (T) and adjacent normal tissues (N) derived from SKCM patients. Data were retrieved from the TCGA database and analyzed by Timer (*p* < 0.05). (**E**,**F**) Human A431 and murine B16F10 cells were cultured with or without indicated concentration of YO-2 for 24 h. Fold change in human and mouse *PANK1* (**E**) or *miR-107* (**F**). Expressions were compared to expression in untreated control cells. (**G**) Fold change in *miR-107* expression determined by qPCR in mock or LRP1 OE cells treated with/without YO-2 (*n* = 3/group). The expression was compared to untreated Mock cell expression. (**H**) Fold change in *miR-103* and *miR-107* expression in miR-103/107 OE compared to its expression in mock B16F10 cells as determined by qPCR (*n* = 3/group). (**I**) Fold change in *LRP1* expression in miR-103/107 OE B16F10 cells compared to mock controls by qPCR (*n* = 3/group). (**J**) si-miR-107 and si-ctrl B16F10 cells were treated with/without YO-2. The percentage of dead cells was determined after 24 h in culture (*n* = 3/group) by trypan blue extinction. (**K**) Fold change in *miR-103, miR-107*, *TP53*, *P21*, *LRP1* expression in tumor tissues of the carrier (PBS) and YO-2-treated mice at day 12 compared to the expression in control tissues taken on day 0 as determined by qPCR (*n* = 3/group). Data are expressed as mean ± SEM (unpaired Student’s *t*-test or one-way ANOVA; * *p* < 0.05, ** *p* < 0.01).

The TP53-regulated miRNAs, miR-103 and miR-107, suppressed LRP1 translation and induced cell death in MEF1 cells [6]. We found that P53 OE B16F10 cells showed enhanced *miR-107* expression, while *miR-107* expression was unchanged in P53KD B16F10 cells compared to control cells (Figure 4A). YO-2 treatment enhanced miR-107 expression in mock and P53 OE, but not P53 KD cells, as we had already determined for p53 expression (Figure 4A). Further analysis confirmed that miR-107 and its host gene PANK1, but not another gene of the PANK family (PANK3; Figure S1A) were downregulated in human and murine melanoma cell lines. In contrast, fibroblastic/stromal and endothelia or multiple myeloma cells highly expressed PANK1 and miR-107 (Figure 4B,C). Supporting our data of miR-107 downregulation, in melanoma cell lines and melanoma tumor tissues of SKCM patients showed lower PANK1 gene expression compared to adjacent normal skin tissues (Figure 4D).

Extending our observation, YO-2 exposure in a dose-dependent manner augmented the expression of *miR-107* and *PANK1* in human A431 and murine B16F10 cancer cells (Figure 4E,F). miR-103/107 OE cells generated by lentivirus showed lower LPR1 expression than control melanoma cells (Figure 4G,H). If YO-2-mediated apoptosis is linked to the restoration of cellular miR-103/107, si-miR-103/107 cells should be resistant to YO-2-induced cell death compared to si-ctrl cells (Figure 4I). Indeed, YO-2 treatment resulted in a lower percentage of dead cells in si-miR-103/107 cells (Figure 4J).

The p53 downstream target and cyclin-dependent kinase inhibitor p21 are critical for p53-mediated G1/S boundary cell cycle arrest and cell senescence [17]. Next, we investigated gene expression in tumor tissues of YO-2-treated B16F10-tumor cell inoculated mice. High expression of *p53*, *p21*, *miR-103*, *miR-107*, but low *LRP1* expression was detected in the collected tumors from mice treated with YO-2, but not in control mice (Figure 4K). These data follow our initial findings that YO-2 enhances p53 and downregulates LRP1 in vitro.

### 3.5. YO-2-Induced miR-107/miR-103 Expression Targets LRP1 in Melanoma Cells

**Figure 5:**
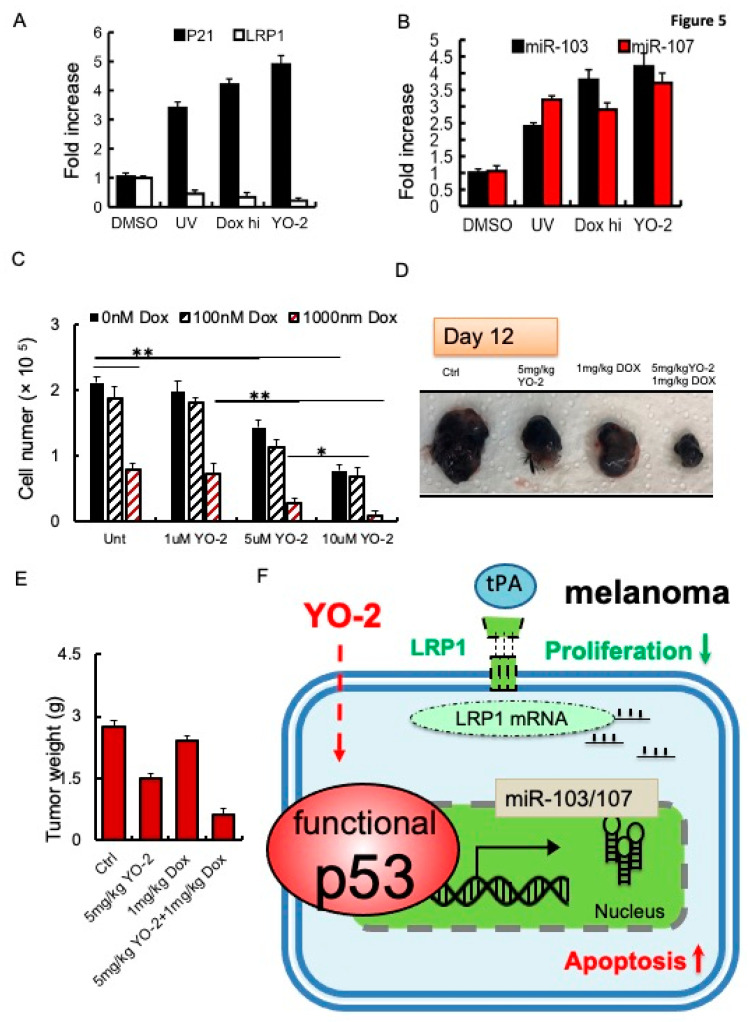
Increased sensitivity of doxorubicin to control tumor growth in YO-2-treated tumors. (**A**,**B**) Fold change in *P21* and *LRP1* (**A**), and *miR-103* or *miR-107* (**B**) expression in UV-exposed, doxorubicin (high)-, and 10 μM YO-2-compared to DMSO-treated B16F10 cells (*n* = 3/group). We normalized most genes to b-actin. mi-103 and miR-107 expression were normalized to U6 gene expression. All data are presented as a relative fold change to controls according to the comparative Ct method (2^−ΔΔCt.^) and represented as mean ± SEM, with *p* values from unpaired Student’s *t*-test. * *p* < 0.05, ** *p* < 0.01. (**C**) The proliferation of B16F10 cells treated with/without YO-2 in the presence/absence of low (100 nM) and high doxorubicin (1000 nM) (*n* = 3/group) as determined by counting cells 24 h after cell plating (*n* = 6/group). (**D**,**E**) B16F10 cells were injected s.c. into the right flanks of C57/BL6 mice. Mice were treated with a single dose of doxorubicin on day 5, and cotreated with/without YO-2 in the indicated groups every other day starting from day 5. Twelve days after injection, tumor weight was measured (**E**; *n* = 6/group), and macroscopic tumor images were taken (**D**). * *p* < 0.05, ** *p* < 0.01 (**F**) Proposed mode of action for YO-2 in melanoma: YO-2 upregulates TP53 (also called p53) upregulating miR-103/miR-107, which targets the LRP1 transcript. Due to the lower LRP1 expression, tPA cannot enhance proliferation in melanoma cells. Abbreviations: LRP1, low-density lipoprotein receptor-related protein 1, miR-103, micro-RNA-103.

Because YO-2 altered the cellular p53 status, we compared p53 downstream target gene expression after YO-2 with other well-known p53 inducers, including UV light and high doses of doxorubicin (1000 nM). Like YO-2, all mentioned p53 inducers upregulated the p53 downstream targets *P21* and *miR-103*, *miR-107* and downregulated *LRP1* expression (Figure 5A,B). While the clinical response of YO-2 could be established in melanoma, the adverse events associated with their on-target effects should be considered. Non-malignant mouse embryonic fibroblasts (MEF-1) and human peripheral mononuclear cells (hPBMCs) treated with 1–10 μM of YO-2 did not reduce the cell numbers or increase the number of dead cells as determined by Trypan blue staining (Figure S1C). We determined the YO-2 toxicity profile using a dosage of 5 mg/kg body weight that reduced melanoma growth and induced p53 in murine models and a dosage of 10 mg/kg body weight of YO-2 in vivo. Dose-limiting toxicities can include severe myelosuppression, measured as the number of bone marrow cells per femur. Injections every other day of YO-2 into mice resulted in pancytopenia only at the 10 mg/kg bodyweight dose of YO-2, similar to a doxorubicin dosage of 5 mg/kg body weight (Figure S1D). These data indicated that YO-2 at 5 mg/kg body weight enhanced intratumoral TP53 expression but did not cause myelosuppression.

To reduce toxicities associated with YO-2, we combined YO-2 with the myelosuppressive agent doxorubicin to suppress melanoma cell growth. In vitro, YO-2 at 5 or 10 μM alone, but not doxorubicin (100 nM), reduced B16F10 cell proliferation by ~30–50%, respectively. Combining high doxorubicin (1000 nM) with YO-2 reduced cell proliferation by ~80–90%, respectively demonstrating a better tumor suppression than either drug administered alone into the cultures (Figure 5C). Tumor growth was blocked in B16F10-tumor-bearing mice cotreated with YO-2 and high-dose doxorubicin, while a single administration of YO-2 or doxorubicin alone only partially suppressed tumor growth (Figure 5D,E). These data imply that YO-2 induces upregulation of p53 and its target genes p21 and miR-103, and miR-107 in vivo (Figure 5F).

## 4. Discussion

The impaired ability to undergo programmed cell death in response to a wide range of external stimuli provides melanomas with a selective growth advantage and arms cells to resist chemotherapy. Earlier studies demonstrated that ApoE and tPA binding to LRP1 drives melanoma cell proliferation and metastasis [2,4]. tPA enhances melanoma growth and metastasis through LRP1 [2,4]. Restoring LRP1 and tPA in the less aggressive, poorly metastatic B16F1 tumor cells enhanced tumor cell proliferation and led to massive lung metastasis in murine tumor models [2]. YO-2 might be effective in preventing melanoma metastasis. But further studies will be necessary to prove this.

The LRP1 ligand ApoE is targeted by miR-199a-3p and -5p, and miR-1908 and the heat shock factor DNAJA4, suppressing LRP1 signaling on melanoma and LRP8 on endothelial cells [4]. ApoE2, but not ApoE4, promotes growth and metastasis via the LRP1 receptor [18].

Mounting evidence suggests that the endocytosis receptor LRP1 is an essential regulator of cell proliferation and apoptosis. Knockdown of LRP1 enhanced neuronal caspase-3 activation and apoptosis. Here we extend our initial studies on the importance of LRP1 for melanoma cell proliferation. We show that enhancing TP53 expression—either pharmacologically via the small molecule YO-2 or through gene overexpression—in melanoma impaired LRP1 expression and inhibited tumor growth. Mechanistically, we demonstrate that TP53 enhances the expression of the PANK1 gene and its endogenous miRNA, the tumor suppressor miR-103/107. In turn, miR-107, by targeting LRP1 impaired melanoma cell proliferation in vitro and in vivo.

The plasmin inhibitor YO-2, but not other protease inhibitors, cause inter-nucleosomal DNA fragmentation and chromatin condensation [13]. However, how YO-2 induces apoptosis has yet to be fully established. We are the first to identify the small molecule YO-2 as a p53 inducer in human and murine melanoma cells that restores miR-103/107 expression, causing tumor cell growth inhibition. In addition, YO-2 enhanced p53 expression, along with that of its downstream target genes PUMA and BAX, and the activation of caspase 3 and 7 in melanoma cells.

We show that p53 induction by YO-2 enhanced miR-103/107 expression, resulting in the downregulation of LRP1. Importantly, YO-2 was ineffective in cells lacking LRP1. Our data are supported by a study on the role of LRP1 in cell death in mouse embryonic fibroblasts [6]. In addition, Leslie et al. reported that lethal doses of stress upregulate p53-regulated miR-103 and miR-107, suppressing LRP1 translation [6]. While the above-mentioned studies support our findings of p53-mediated miR107 induction with consecutive LRP1 downregulation and melanoma growth inhibition, it will be left for future studies to delineate the molecular mechanism of p53 induction by YO-2.

Our data align with earlier reports demonstrating miR-107 downregulation in melanoma cells and tumor tissues [9]. In melanoma, miR-107 functions as a tumor suppressor [9] by binding in the 3’UTR of POU3F2, a transcription factor regulating melanoma cell invasiveness and proliferation [19]. Interestingly, miR-107 can also target DICER1, a key regulator of miRNAs in differentiated neuronal cells [20]. Dicer and Drosha are the miRNA processing enzymes required for miRNA maturation. Of interest, DICER is upregulated in patients with cutaneous melanoma [21]. Therefore, it will be essential to understand if YO-2 targets Dicer1 in cancer cells.

In non-malignant cells like podocytes, YO-2 inhibited apoptosis and inflammatory/profibrotic cytokines by suppressing the attachment of plasmin(ogen) to podocytes. It was shown that YO-2 suppressed the upregulation of protease-activated receptor-1 (PAR1) [22]. Earlier studies demonstrated that PAR1 is a rate-limiting factor for experimental melanoma lung metastasis [23]. In future studies, it will be important to determine whether YO-2 can suppress melanoma lung metastasis via PAR1 downregulation rather than via p53 induction. However, it is possible that YO-2 targets more than one “unwanted” tumor-promoting factor. The YO-2-mediated antiproliferative and pro-apoptotic effects seem cell-type specific, as YO-2 did not block multiple myeloma growth in murine multiple myeloma model mice in vivo [11]. The TP53 status of cancer cells may be critical. In melanoma, wild-type P53 is present in over 80% of melanomas, but it is largely inactivated. Our data indicate that YO-2 could not induce apoptosis in cell lines such as K562 with known mutant TP53. In multiple myeloma, by contrast, the frequency of mutations and deletions is more frequent in the late stages of the disease and is associated with treatment resistance [24].

Our study demonstrated that the TP53 inducer YO-2, combined with the myelosuppressive drug doxorubicin, controls tumor growth better than either drug given alone. TP53 mutations are frequent, representing a fundamental advantage during cancer development by depriving cells of tumor-suppressive responses, such as senescence and apoptosis.

## 5. Conclusions

It is noteworthy that many studies have reported on the wild-type status of TP53 in melanoma, but only a few studies found a link between the endocytosis receptor LRP1 and TP53. Several lines of evidence demonstrate the encouraging outcomes achieved with p53-activating drugs, alone and in combination with currently available therapies for melanoma patients. We identified YO-2 as a TP53 inducer in melanoma. Our results uncover a novel regulatory circuit driving melanoma growth (Figure 5F): Induction of p53—as achieved in this study by YO-2 treatment—increases the expression of the critical tumor suppressor miR-107, a miRNA that under non-malignant conditions keeps LRP1 expression, and thereby cell proliferation, at bay.

## Data Availability

Not applicable.

## Supplementary Materials

The following supporting information can be downloaded at: https://www.mdpi.com/article/10.3390/cancers15010288/s1, Figure S1: Low PANK1 and miR107 expression in murine melanoma cells.
