# Supplementary material for: YO2 Induces Melanoma Cell Apoptosis through p53-Mediated LRP1 Downregulation
Source: Cancers (Basel). 2022 Dec 31;15(1):288. doi: 10.3390/cancers15010288 (PMC9818169; doi:10.3390/cancers15010288)
Supplement: Supplementary file 1 [file cancers-15-00288-s001.zip › cancers-2092191-supplementary.pdf]

Figure S1

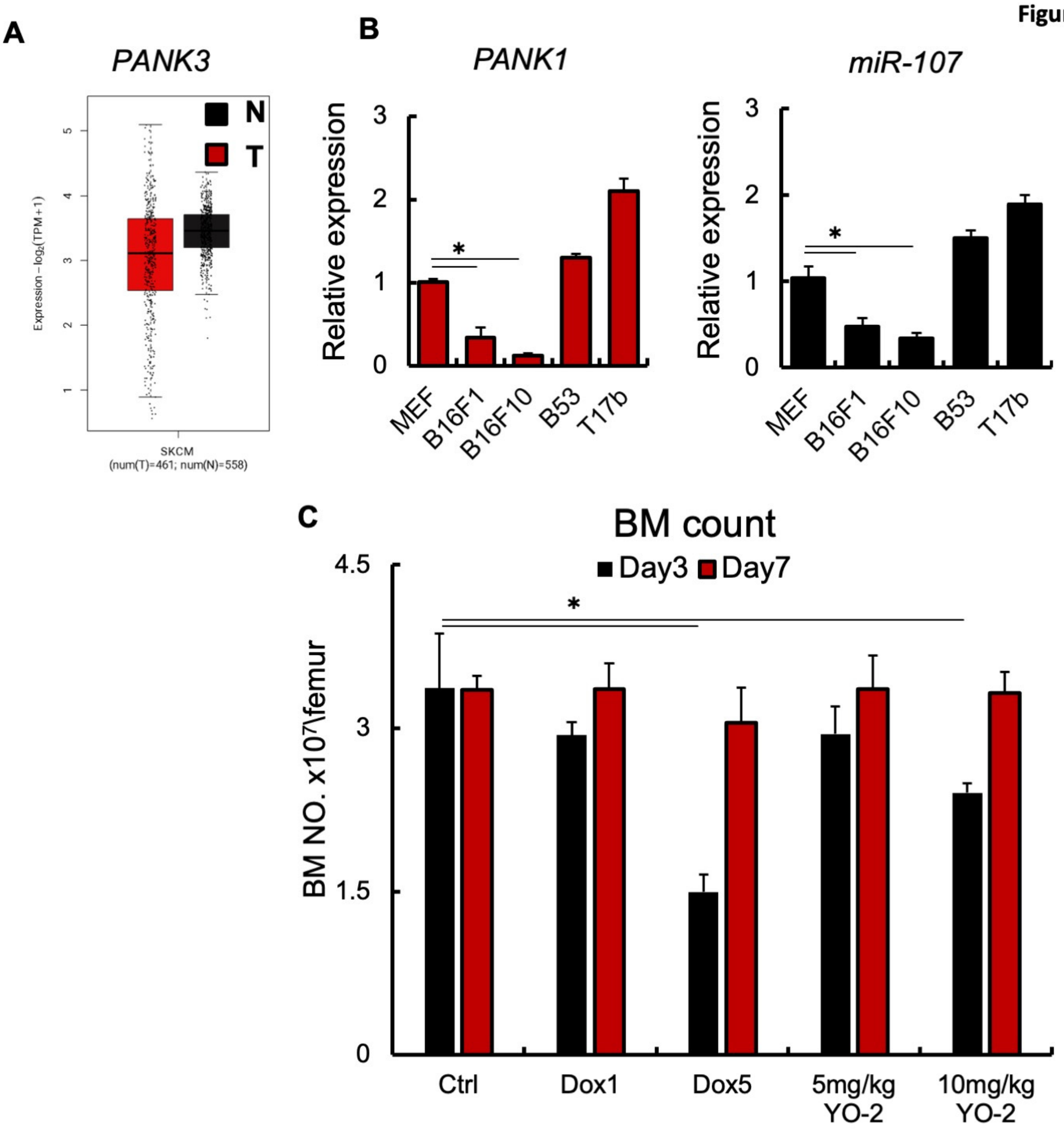

Figure S1. Low *PANK1* and *miR107* expression in murine melanoma cells. (A) Human *PANK3* expression levels in tumor (T) and adjacent normal tissues (N) derived from SKCM patients. Data were retrieved from the TCGA database and analyzed by Timer (n.s.). (B and C) Fold change in mouse *PANK1* (B) and *miR-107* in indicated cell lines compared to expression in MEF cells as determined by qPCR (n=3). (D) Mice were injected with 5mg or 10mg/kg bodyweight YO-2 every other day starting from day 0. Other mice were injected on day 0 with doxorubicin 1mg/kg or 5mg/kg body weight as a single injection. Viable bone marrow mononuclear cells were counted in treated and untreated mice on days 3 and 7 after treatment (n=6/group).

* $p < 0.05$.
